# Computational Design of a Molecularly Imprinted Polymer for the Biomonitoring of the Organophosphorous Metabolite Chlorferron

**DOI:** 10.3390/bios11060192

**Published:** 2021-06-10

**Authors:** Bakhtiyar Qader, Issam Hussain, Mark Baron, Rebeca Jiménez-Pérez, Guzmán Gil-Ramírez, Jose Gonzalez-Rodriguez

**Affiliations:** 1 Sulaimani Medicolegal Institute, Qanat Street, Sulaimani, Sulaymaniyah 46001, Iraq; bakhtyar88@gmail.com; 2 Joseph Banks Laboratories, School of Chemistry, University of Lincoln, Lincoln LN6 7DL, UK; mbaron@lincoln.ac.uk (M.B.); Rebeca.Jimenez@uclm.es (R.J.-P.); GGilramirez@lincoln.ac.uk (G.G.-R.); 3 School of Life Sciences, University of Lincoln, Brayford Pool, Lincoln LN6 7TS, UK; ihussain@lincoln.ac.uk; 4 Department of Physical Chemistry, Higher Technical School of Industrial Engineering, University of Castilla-La Mancha, Campus Universitario s/n, 02071 Albacete, Spain

**Keywords:** environmental analysis, coumaphos metabolite, electrochemical detection, bee pesticide, biomarker

## Abstract

Coumaphos is an organophosphorus compound used as insecticide and frequently used by beekeepers for the management of parasitic mites. The most important metabolite, chlorferron (CFN), has been identified in biological samples and foodstuff. The need to quickly identify the presence of typical metabolites, as an indication of interaction with coumaphos has driven the need to produce a highly sensitive electrochemical method for chlorferron analysis, based on molecularly imprinting polymers (MIP) technology. It showed irreversible behaviour with mixed diffusion/adsorption-controlled reactions at the electrode surface. A monoelectronic mechanism of reaction for oxidation has also been suggested. The linear range observed was from 0.158 to 75 µM. Median precision in terms of %RSD around 3% was also observed. For DPV, the limit of detection (LOD) and the limit of quantitation (LOQ) for the CFN-MIP were 0.158 µM and 0.48 µM, respectively. The obtained median % recovery was around 98%. The results were also validated to reference values obtained using GC-MS. Urine and human synthetic plasma spiked with CFN were used to demonstrate the usability of the method in biological samples, showing the potential for biomonitoring. The developed imprinted sensor showed maximum signal change less than 16.8% when related metabolites or pesticide were added to the mix, suggesting high selectivity of the MIP sensor toward CFN molecules. The results from in vitro metabolism of CMP analysed also demonstrates the potential for detection and quantification of CFN in environmental samples. The newly developed CFN-MIP sensor offers similar LoDs than chromatographic methods with shorter analysis time.

## 1. Introduction

Coumaphos [*O*,*O*-diethyl *O*-(3-chloro- 4-methyl-2 oxo-2H-1-benzopyran-7-*yl*) phosphorothioate (CMP)], is an organophosphorus compound used as a pesticide and was first used in 1958.

**Figure 1:**
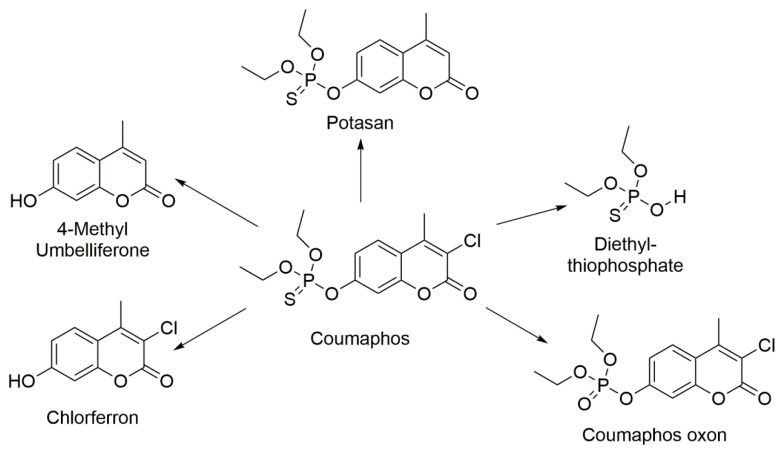
Coumaphos and its metabolic products.

Several metabolites including potasan, chlorferron, 4-methylumbelliferone, coumaphos oxon, and diethyl thiophosphate have been identified in human urine, bee products, water, and food samples by different studies [1,2,3,4] (Figure 1). 3-chloro-4-methyl-7-hydroxycoumarin (Chlorferron, CFN) is one of CMP major metabolites and structurally related to 4-methylumbelliferone (4-MU). In addition, CFN was observed as a metabolite of CMP in samples from children exposed to CMP residues.

In general, the absorption of CMP is mainly through skin depending on the animal species. Additionally, CMP poisoning may occur through oral route. Following its ingestion is absorbed into the blood and rapidly metabolised by liver enzymes and distributed to other tissues, particularly muscle, fat tissues, and milk. Finally, the metabolites are excreted through urine and part of it was excreted unchanged in the stool.

Typical analytical techniques used for coumaphos analysis and following the fate of metabolites in different matrices involved the use of fluorometric methods [1,2,3], High Performance Liquid Chromatography (HPLC-DAD) [4,5,6], Gas Chromatography (GC) [7,8,9,10], and Liquid Chromatography (LC-MS) [11,12].

The absence or presence of the main pesticide molecule and its metabolites in biological specimens can be of use to identify recent exposure to the pesticide [13,14,15]. Measurements of specific biomarkers of the pesticide in human urine is important to assess toxic risks associated with the pesticide exposure; for instance, CNF is a biomarker for CMP exposure [6]. Hence, HPLC hyphenated to mass spectrometry was used for the identification of characteristic metabolites, used as biomarkers, in human urine. In this method, CFN was quantified as biomarker for CMP along with other metabolites for some other pesticides with LOD as low as 0.2 ng/mL of CFN in urine [6].

In this paper, we performed a study of the electrochemical behaviour of coumaphos main metabolite, chlroferron (CFN), for its identification and quantification in biological samples. In order to enhance the sensitivity and selectivity of the sensor, a glassy carbon electrode was electropolymerised with a molecularly imprinted polymer (MIP). To date, no MIP based on electrochemical or other detection method has been reported for CMP metabolites or CFN, in particular. In this study, computational calculations were also implemented (DFT-B3LYP with 6-31G), which allowed the selection of the most suitable monomer of those used in the literature. We also obtained the best value for the monomer-template using semi-empirical computational analysis using PM3.

## 2. Materials and Methods

### 2.1. Chemical and Reagents

Coumaphos, 3-chloro-4-methyl-7-hydroxycoumarin (Chlorferron), and 4-methylumbelliferone were ordered from Sigma (Sigma-Aldrich, Leicestershire, UK). Acids used in this work were: hydrochloric, phosphoric and glacial acetic acids and were purchased from Fisher Scientific (Leicestershire, UK). Disulfoton-sulfoxide (DSX) and propoxur (PPX) were also delivered by Sigma-Aldrich and used for testing the selectivity of fabricated GC electrode with electro-polymerisation of pyrrole. Potassium hydroxide was also obtained through Fisher Scientific (Leicestershire, UK). Sodium chloride was acquired from Sigma-Aldrich, UK. Britton-Robinson buffer was freshly prepared every day, and the pH was adjusted as required to obtain the different pHs used in this work. Potassium ferricyanide was purchased from Sigma-Aldrich (Leicestershire, UK) and helped in assessing the state of the electrode surface. Fumed silica (0.007 μm) and Al_2_O_3_ (0.05 μm) used to polish the working electrode were both from Sigma-Aldrich (Leicestershire, UK). Acetonitrile (HPLC grade) was obtained from Fisher (Fisher Scientific, Leicestershire, UK). Deionised water (18 MΩ) was used to prepare all solutions. Artificial human plasma was obtained from Sigma Aldrich, UK. Urine samples were obtained from healthy volunteers.

The reagents used for the metabolic system can be found as described by Qader [16].

### 2.2. Instruments and Apparatus

A Metrohm 757 VA Computrace system (Metrohm Ltd., Runcorn, UK), was used to perform all the electrochemical procedures. A three-electrode configuration was used, where a glassy carbon (GC) electrode was selected as a working electrode, a Ag/AgCl in 3M KCl electrode was used as a reference electrode, and a platinum electrode as counter-electrode. A Hanna instrument microprocessor pH 210 m1ter pH-meter was used to monitor pH. An ultrasound bath (Kerry, UK) used to ultrasound treatment was also used.

The Gas Chromatography-Mass Spectrometry system used for these analyses was a Perkin Elmer GC model Clarus 500. The column installed was a SUPELCO analytical, SLB-5 m fused silica capillary column (30 m × 0.25 mm × 0.25 μm).

Stirring bath used in the metabolic analyses was from Grant Instruments (Cambridgeshire, UK). MiKro 20 Hettich Zentrefugen Microcentrifuge (Beverly, CA, USA); Electric balance from Sartorius (Göttingen, Germany), Parafilm paper, Eppendorf tube and racks, auto pipettes, cuvettes, and potable colorimeter set to 590.

### 2.3. Solution Preparation

Standard solutions (30 mM) of CMP, CFN, and 4-MU were prepared and stored at 4 °C in amber bottles. Diluted solutions for this work were prepared daily using the Britton-Robinson buffer (BR buffer). One litre of 0.5 M BR buffer was prepared by adding 33.78 mL of phosphoric acid (14.8 M) to 28.6 mL of acetic acid (17.48 M) and 29.22 g of NaCl and mixed with distilled water.

A polymerisation solution to functionalise the electrode was prepared by mixing 4 mM pyrrole and 1 mM CFN in 100 mM BR buffer (pH, 7) solution. The extraction solution for removal of the CFN from the molecularly imprinted polymer was prepared by using 2 mL of acetic acid and 10 mL of acetonitrile.

### 2.4. Experimental Procedures

All working electrodes were polished using aluminium oxide mixed with water to create a paste and used first to create a mirror-like surface and a silica paste used after to smooth it. The electrodic surface was then rinsed with water and then submerged in acetonitrile for 5 min in an ultrasonic bath. All experiments were performed using a 50-mL cell at 25 °C after purging with N_2_ gas for 5 min. The electrode was tested using a 0.01 M K_3_[Fe(CN)_6_] solution.

The electro-polymerisation was conducted in solution containing 4 mM pyrrole, 1 mM CFN, and 100 mM BR buffer at pH, 7 after bubbling N_2_ for five minutes. The polymerisation involved the use of a cyclic voltammetry method in a potential range of −0.6 V to +1.0 V (vs Ag/AgCl) at scan rate = 0.1 Vs^−1^ for 5 scan cycles. The NIP was constructed in the same fashion without the presence of CFN.

The CFN molecules in the MIP were removed from the polymeric film by immersing the MIP electrode into a stirred mixture of acetic acid and acetonitrile at a ratio of 2:5 (*v/v*). Finally, the molecularly imprinted GC electrode was then dried by blowing under nitrogen gas.

Analytical determination of CFN was performed by cyclic voltammetry (CV) using a method using potentials starting at 0.3 to 1.3 V using a scan rate of 100 mV s^−1^ and a equilibration time of 10 s. Differential pulse voltammetry (DPV) was performed in the same potential range with a voltage step of 9.918 mV, pulse amplitude of 50 mV, a pulse time of 0.04 s, voltage step time of 0.4 s and a sweep rate of 0.0248 Vs^−1^.

Synthetic human plasma (one millilitre) was diluted with 29 mL of B-R buffer at pH, 2. Additionally, for the analysis of urine, two millilitres of fresh urine were diluted to 30 mL with the BR buffer solution.

GC-MS method used was as follows: Starting temperature 100 °C, stand for a minute, ramp 35 °C min^−1^ until 170 °C, stand for 1 min, ramp 15 °C min^−1^ to a final temperature of 290 °C, stand for 4 min. Carrier gas was helium at a flow rate of one mL min^−1^ and the injection volume was 1 μL. The temperature of the transfer line was set at 280 °C. A positive ionization mode, using electron impact, and keeping the source at 260 °C was performed with an electron energy of 70 eV and the multiplier set at 350 V. Two mins solvent delay was applied to achieve a total an analysis time of twenty minutes.

Incubation experiments were conducted as described by Qader [16] with the only modification of using coumaphos as starting molecule to produce the metabolite chlorferron.

### 2.5. Computational Approach

All computational calculations were performed using Spartan software (Wavefunction). DFT-B3LYP with 6-31G was used, which allowed the selection of the most suitable monomer of those used in the literature. Template-monomer molar ratio was computationally optimized using Semi-empirical (PM3) calculations. All calculations were performed in a vacuum. One of the most promising computer-based methods available to assist in MIP design is simulation via molecular dynamics. While quantum and semi-empirical approaches allow determination of the absolute energies of monomer template interactions, these methods tend to be limited by a number of factors such as the inability to explicitly simulate solvent or to account for dimerisation of mixture components. Molecular dynamics studies, in contrast, permit the qualitative evaluation of the types of interaction occurring in a mixture, the frequency of such interactions across a given time frame and the lifetimes of complexes. DFT and semi-empirical approach were selected in this paper, as no solvent was considered in the calculations, as the computational approach was used as a screening for the best monomer.

## 3. Results and Discussion

### 3.1. Glassy Carbon Analysis of CFN

**Figure 2:**
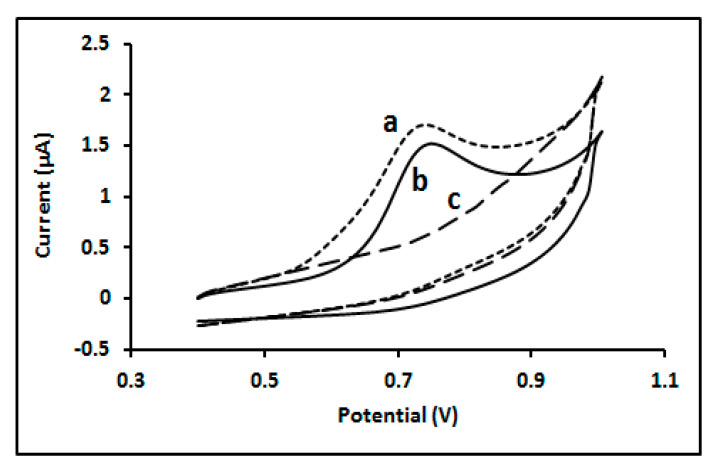
Cyclic voltammogram of (**a**) 50µM 4-MU; (**b**) 50µM CFN; (**c**) in absence of both analytes; at glassy carbon electrode in 0.1 M BR buffer (pH, 7) solution.

The voltametric behaviour of the major metabolites of CMP, CFN and 4-MU, were studied using a bare GC electrode by CV. These two metabolites showed good electroactivity on a carbon electrode. The two metabolites have shown approximately the same voltametric behaviour when studied at pH = 7. A buffered solution containing CFN and another one containing 4-MU rendered only one anodic peak observed at potential of 0.736 V for CFN and 0.726 for 4-MU, respectively, when cyclic voltammetry at a scan rate of 100 mV s^−1^ was used. No peaks were detected in the reverse scan, indicating the reaction processes are irreversible in both metabolites, as seen in Figure 2.

**Figure 3:**
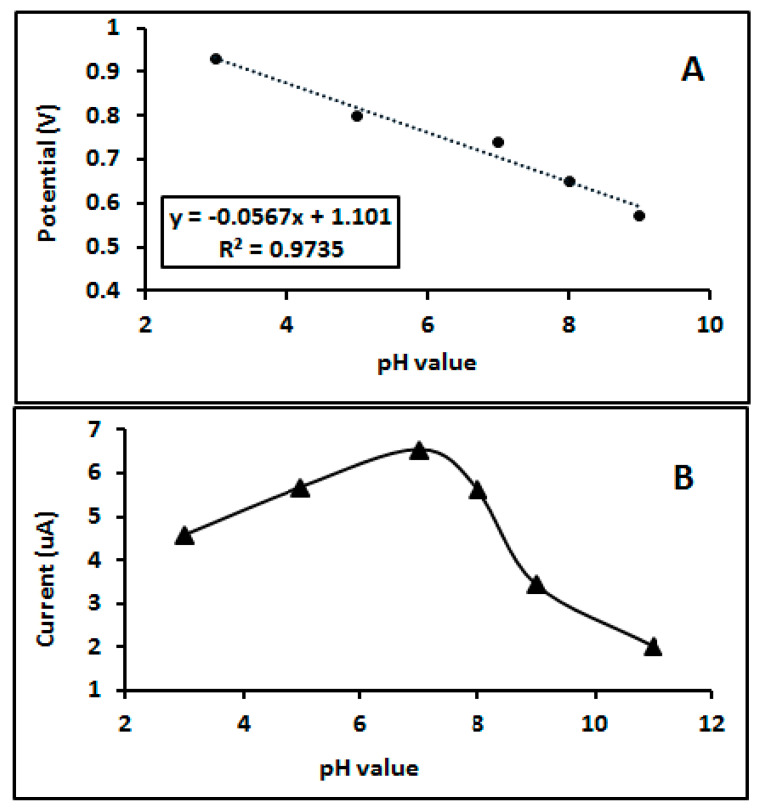
(**A**) Influence of pH on Potential peak; (**B**) influence of pH on current response; of 50 µM CFN in 0.1 M BR buffer on a bare GC electrode.

It was clear that pH played a role on the oxidation of CFN, as demonstrated by a DPV study where pH was changed from 3 to 9. An increase of pH rendered increments in the peak potential (Figure 3A), according to the following equation:

Ep = 0.0567 pH + 1.101, r^2^ = 0.9735 (1)

The linearity changes between peak potential and pH of analysed media ensuring that protonation is participating in the overall reaction taking place at the electrode surface [17,18,19,20]. In addition, the peak current intensity changed with the changing pH of the media and the optimum intensity was obtained at pH 7 as seen in Figure 3B.

More information about the electrochemical mechanism of the reaction and the influence of the scan rate (υ) on the CFN oxidation was obtained by analysing cyclic voltammograms in the range 50–1000 mV/s (Figure S1). The potential shifted towards positive values with increasing scan rates, thus confirming the irreversible nature of the oxidation reaction.

To evaluate the nature of the control process for the reaction occurring at the electrode and whether this was either adsorption or diffusion controlled, a plot of square root of can rate and peak current intensity (I_p_) was performed. Figure S2 showed a linear relationship between scan rate and current response, suggesting the reaction is diffusion controlled [21].

Additionally, the logarithm of peak intensity (log I_p_) was plotted vs. the logarithm of scan rate (log υ) offering a linear response, as expressed by the equation:

Log I_p_ = 0.7677 log υ + 1.5706, r^2^ = 0.9964 (2)

As the slope gives a value of 0.76, located between 0.5 (diffusion control) and 1 (adsorption control), we concluded that the reaction follows a mixed adsorption–diffusion controlled process [19,22].

The number of electrons involved in the electrochemical process was also assessed and a plot of peak potential (E_p_) versus logarithm scan rate (log υ) was performed in a sweep rate range 50–1000 mV/s. This rendered a linear relationship following the equation:

Log E_p_ = 0.1016 log υ + 0.7971 (3)

According to Laviron’s equation for irreversible species,

(4) $$E_{p}=E{}^{\circ}+\left(\frac{2.303\mathrm{RT}}{\alpha_{n}F}\right)\log\left(\frac{\mathrm{RTK}{}^{\circ}}{\alpha_{n}F}\right)+\left(\frac{2.303\mathrm{RT}}{\alpha_{n}F}\right)\log ʋ$$

The slope of E_p_ vs. log υ informs of the transfer coefficient of the process (α_n_*)*, and we can calculate it using the equation. From the calculation, α_n_ is equal to 0.57 with T = 298 K, R = 8.314 J/K mol and F = 96,480 C/mol.

**Figure 4:**
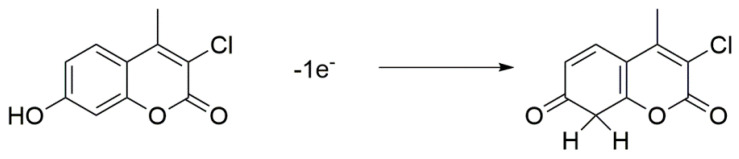
Mechanism of CFN oxidation at GC electrode.

Additionally, based on Bard and Faulkner equation (2002), α can be calculated from this equation:

α *=* 47.7/*E_p_* − *E_p_*_1/2_ (5)

where *E_p_*_1/2_ is the half wave potential. Therefore, from this we got obtain a value for *α* of 0.53, close to the previous one. Further, the number of electrons (n) shared in oxidation reaction of CFN is equal to 1.07 ≈ 1. Therefore, the proposed mechanism of oxidation reaction of CFN occurred on the electrode surface is suggested as seen in Figure 4.

### 3.2. Computational Approach

After the preliminary studies to assess the viability of building a molecular imprinted polymer for CFN, the next stage will involve the calculation of the theoretical binding energies between the selected monomers and the template using DFT for the best functional monomer.

This first step in the production of a MIP will inform of the formation of a stable system between the target molecule (CFN) and the polymer. Therefore, selecting the most suitable functional monomer is very important for the MIP design [23]. A model based on a DFT approach at B3LYP/6-31G level was performed to assess the conformational optimization of CFN and eight potential suitable functional monomers (aniline (A), 2-aminophenol (OAP), ethelenedioxythiophen (ETOP), N-isopropylacrylamide (IPA), 2-Mercaptobenzothiazole (MBT), naphthalenedisulfonic acid (H-A), 0-phenelendiamine (OPD), and pyrrole. All of them can produce conductive polymers suitable for electrochemistry on a glassy carbon electrode. The computational procedure will screen the stability values and offer an estimate of the binding energies. It is important to highlight that the results do not offer an accurate picture of the real values expected, as many assumptions are made, but it does offer some indication of the most suitable. Calculated energies (E) are shown in Table 1. The binding energy of the template-monomer system, ΔE, was estimated using Equation (6) [24]:

ΔE = E(template–monomer) − E(template) − ∑E (monomer)(6)

According to the results achieved, the best monomer for preparing the MIP should offer the highest level of stability to enhance the selectivity. According to this, CFN-Py was shown to be the best candidate for the design and build of the sensor. The next monomer offering the second more stable configuration, ethelenedioxythiophen (ETOP), also offers a very good value. As the aim of the paper was not to establish a comparison between the two potential polymers, which could certainly be interesting, we followed the lowest as given by CFN-Py.

### 3.3. Fabrication of the CFN Imprinted Sensor

The MIP was prepared using a procedure based on Mamo and Gonzalez-Rodriguez [25] as shown in the experimental section.

**Figure 5:**
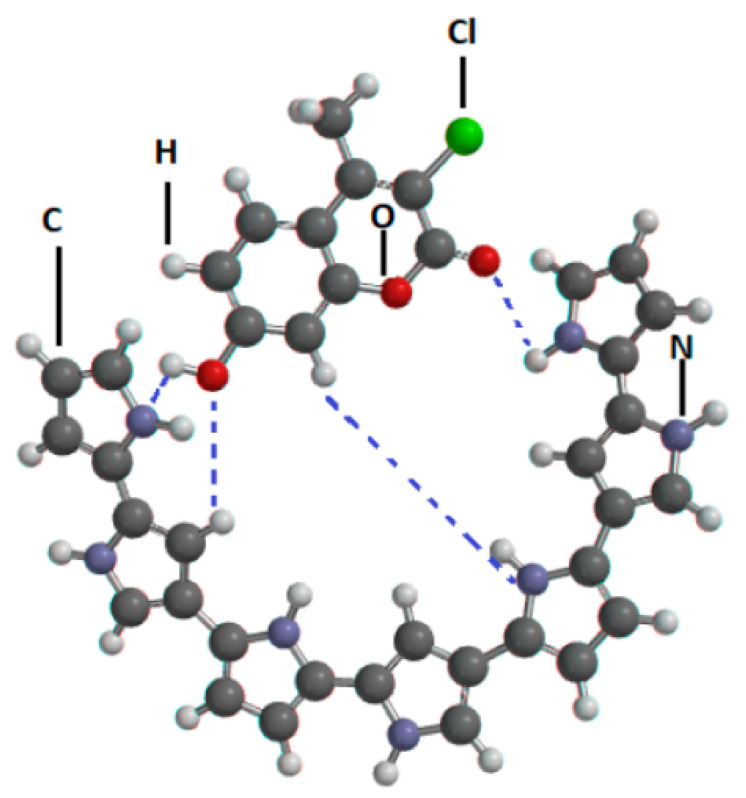
Representation of the CFN-Polypyrrole interaction during electro-polymerisation. (N = Nitrogen, O = Oxygen, Cl = Chlorine, C = Carbon, H = Hydrogen and **- - -** = hydrogen bond).

It is important to highlight the reversible nature of the interactions between the template molecule and the polymer network during the imprinting process. This is paramount to obtain a sensor system that can be reused. The oxygen in the CFN molecules will interact with hydrogens present in the hydroxyl groups of the polymer through hydrogen bonding, but also the nitrogen in the pyrrole structure will follow the same sort of interaction with the acidic protons in the structure of chlorferron (Figure 5).

### 3.4. Optimisation of the Imprinted Sensor

The importance of optimization factors was mentioned in the method section. CFN-Py molar ratio was also optimized using computational methods by implementing a semi-empirical (PM3) calculation. The results of these calculations resulted in the selection of a CFN:Py (1:4) ratio as the optimum to trap the CFN within the polymer structure. Therefore, the electro-polymerisation was performed with this ratio.

Additionally, it is important to optimize the number of cycles used in the production of the polymeric film to achieve maximum number of cavities and good conductivity, avoiding excessive resistivity. The value found to produce the highest current in this system was of five scan cycles. The optimization process will maximise the number of cavities, hence the LoD. However, other factors in the real sample can affect whether these cavities will trap the sample effectively, reducing the real sensitivity of the MIP produced (i.e., sample matrix, ionic strength, pH). Different compounds and different MIPs and samples will produce different LoDs, as it is well illustrated by Chen et al. in their review on MIPs [26]

### 3.5. CFN-MIP Sensor Voltammetry-Performance

**Figure 6:**
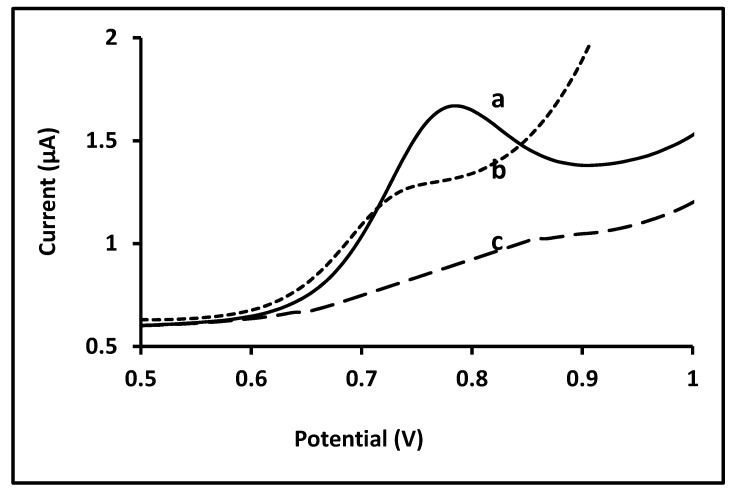
Differential pulse voltammetry of 10 µM CFN in 0.1 M BR buffer solution (**a**) on CFN-MIP sensor after removal step; (**b**) on bare GC electrode and (**c**) NIP electrode.

The responses obtained with the bare glassy carbon electrode and the imprinted electrode to CFN were also compared using DPV. The potential peak of CFN was shifted to positive direction compared to that on the bare GC electrode due to formation of a modified surface on the glassy carbon (MIP). In addition, the CFN current intensity (µA) on the MIP offers a higher value than that obtained for the glassy carbon electrodic surface (note: consider the different baselines). This clearly shows a better performance of the polymer electrode when the CFN-MIP is compared to the GC electrode. Additionally, the response of the NIP to CFN is clearly negligible (Figure 6).

**Figure 7:**
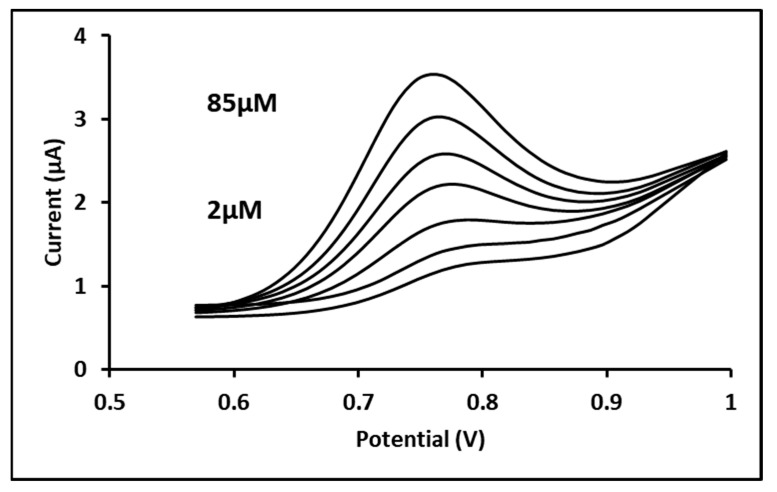
Differential pulse voltammogram for seven concentrations of 2, 10, 25, 40, 55, 70, and 75 µM CFN in 0.1 M BR buffer (pH = 7) solution on CFN-MIP electrode; Experimental conditions: pulse amplitude, 50 mV; pulse time, 0.04 s; and sweep rate, 25 mV/s.

The response of the developed MIP sensor was also studied in BR buffer at pH = 7; the peak current was increased with increasing concentrations of CFN in the solution when monitored by DPV as seen in Figure 7. The response of the electrode to increasing concentrations of CFN offered a lineal range in the studied range.

For DPV, the Limit of Detection (LoD) and the limit of quantitation (LoQ) for the CFN-MIP were 0.158 µM and 0.48 µM, respectively. As a comparison, the LoD and LoQs obtained for the analysis of CFN using the glassy carbon electrode were 0.91 µM and 2.78 µM, respectively, suggesting a higher sensitivity for the CFN-MIP.

Furthermore, the sensitivity of the proposed EC method is comparable to the HPLC electrospray ionization–tandem mass spectrometry reported for the analysis of FNP in human urine [6].

The precision of the CFN-MIP was assessed by calculating the %RSD for five measurements on the same day (intra-day precision) and the %RSD determination of five consecutive days (inter-day precision). The precision was performed using seven CFN concentrations with DPV, as shown in Table S1, in Supplementary Materials. As a summary, the calculated precisions were in the range 2.35–8.75% and 3.22–13.1% for intra-day and inter-day, respectively. In addition, the precision of the determinations for intra-day concentrations is better than those for inter-day concentrations, as expected. Overall, the median precision is around 3% for both intra and inter-day measurements.

The recovery rate was evaluated using seven concentrations of CFN prepared from a standard stock solution in triplicates by DPV as shown in Table S2, in the Supplementary Materials. Thus, the median recovery is around 98%. In order to validate the polymer sensor results, the same concentrations of CFN (25, 40, 55, 70, and 85 µM) were also injected in the Perkin–Elmer gas-chromatograph mass-spectrometry system and the values obtained used as a reference. The direct comparison of the results when plotted depicted a straight linear relationship with a slope close to 1 and a slight deviation on the intercept (GC-MS = 0.9972 CFN-MIP + 1.6067) between the recovered concentrations as shown in Figure S4, in the Supplementary Materials.

The selectivity of the CFN-MIP sensor in the presence of four interferences was also studied. Selected compounds were: propoxur (PPX), disulfoton-sulfoxide (DSX), coumaphos (CMP), and 4-ethylumbelliferone (4-MU). These were chosen because they are chemically related, as in the case of 4-MU, or they are other organophosphorus pesticides. The influence of different concentrations (up to 10 fold) of each substrate in the analysis of a 10 µM CFN solution was investigated, and the results shown in Table 2.

It can be observed that the level of interaction increases with the concentration of the interference. In all cases, this is never greater than 16.88% of the CFN signal, for the case of disulfoton-sulfoxide. A very interesting case is that of 4-MU, as this is another of the coumaphos metabolites. It can be seen that in the worst case scenario, a tenfold excess concentration of 4-MU only disrupts CFN measurement by 10.82%. This is an indication that the selectivity of the imprinting methodology is very good and can only recognize the CFN mainly.

### 3.6. Application for Biological Samples

The applicability of the CFN-MIP sensor to biological samples and a microsome metabolic system were also evaluated in this paper. Samples of synthetic human plasma and urine samples were spiked with known amounts of CFN. Two different concentrations of CFN (25 and 40 µM) were added to the plasma and urine samples before being measured by DPV using the CFN-MIP sensor. Table 3 shows the results of these analyses. The recovery rate achieved was in the range 91.61–98.19% and the %RSD value is less than 8.88%. The results obtained from our study evidenced the lack of significant influence the matrix exerted on the recovery of the analyte, showing the high selectivity of the CFN-MIP sensor. When comparing the values obtained for both urine and plasma, the matrix effect observed was slightly lower for the former.

The electrochemical methods developed in this study were tested for the determination of chlorferron in samples obtained from an in vitro study of the coumaphos metabolism. A thousand µL from a solution produced in the in vitro metabolism was evaporated and then reconstituted with 1 mL B-R buffer (pH = 7) solution. The sample specimens were measured by DPV using the MIP-CFN electrodes in triplicate. Results are shown in Table 4.

## 4. Conclusions

A highly reproducible and sensitive polymeric sensor for the determination of CFN in biological samples has been successfully produced. The CFN molecule showed electroactivity on a bare glassy carbon electrode using CV and DPV. One oxidation peak was noted and has optimum peak intensity at pH 6. The peak showed irreversible behaviour with mixed diffusion–adsorption controlled reaction at electrode surface, proved from experiments at various scan rates. As well as from scan rate experiments, a proper mechanism of reaction for oxidation of CFN was suggested based on calculation of the number of transferred electrons in the system; which was one electron.

An MIP sensor was designed using computational methods with pyrrole as the most suitable monomer. The concentration of template CFN to pyrrole ratio in the polymeric mixture was 1:4 mole calculated by a semi-empirical computational method. Moreover, a simple and efficient MIP based EC sensor for CFN was prepared by 5 cycles electro-polymerisation. The linear range was from 0.158 to 75 µM, with a CV LoD of 0.158 µM. The method is suitable to follow the bioconversion of coumaphos to chlorferron. Compared to the bare glassy carbon electrode, the sensitivity of the imprinted sensor showed greater improvement. Repeatability of the CFN-MIP sensor was less than 8.88% with a recovery percentage of greater than 91.61% in spiked urine and plasma samples.

The developed imprinted sensor showed maximum signal change less than 16.8% in presence of potential structurally related interferents, suggesting high selectivity of the CFN-MIP sensor against CFN molecules. The results from in vitro metabolism of CMP explained that EC methods can be used for detection and quantification of CFN in real samples.

## Figures and Tables

**Table 1:** Calculating binding energy of CFN and monomers using DFT model at B3LYP level.

Monomers	E (CFN-Monomer) (KJ/Mol)	E(CFN) (KJ/Mol)	E(Monomer) (KJ/Mol)	ΔE (KJ/Mol)
MBT	−2192.04511	−1071.15051	−1120.88752	−0.00708
ETOP	−1852.002872	−1071.15051	−780.840446	−0.011916
Py	−1281.328453	−1071.15051	−210.165882	−0.012061
H-A	−2704.622198	−1071.15051	−1633.46655	−0.005138
OPD	−1414.110633	−1071.15051	−342.955368	−0.004755
IPA	−1436.397477	−1071.15051	−365.235993	−0.010974
A	−1358.76093	−1071.15051	−287.601785	−0.008635
OAP	−1433.979616	−1071.15051	−362.818674	−0.010432

**Table 2:** Effect of interferents on DPV current response of anodic peak of 10 µM CFN at CFN-MIP electrode.

Interferent Molecule	Concentration (µM)	Signal Change	Signal Percent Change (%)
4-Methyl umbelliferon	10	0.004	1.73
	50	0.03	12.98
	100	0.037	16.01
Coumaphos	10	0.009	3.89
	50	−0.007	3.03
	100	−0.025	10.82
Disulfoton -sulfoxide	10	−0.001	0.43
	50	−0.02	8.65
	100	−0.039	16.88
Propoxur	10	−0.002	0.86
	50	−0.023	9.56
	100	−0.024	10.38

**Table 3:** Values obtained for the recovery analysis of spiked urine and plasma samples when known concentrations of chlorferron were analysed by DPV using a CFN-MIP sensor.

Interference Media	Concentration Spiked (µM)	Mean (µM)	Recovered Percentage (%)	RDS (%) (N = 3)
Plasma N = 3	25	22.9	91.61%	6.58%
	40	38.98	97.45%	3.84%
Urine N = 3	25	23.71	94.84%	8.88%
	40	39.27	98.19%	7.81%

**Table 4:** Values obtained from the DPV CFN-MIP analysis of two specimens obtained from the in vitro metabolism of coumaphos to produce chlorferron.

Electrochemical Method	Sample ID	CFN Mean (µM)	RSD (%) (N = 3)
DPV-CFN/MIP	A	4.54	4.56
	B	7.41	6.41

## Data Availability

The data that support the findings of this study are available from the corresponding author upon request.

## Supplementary Materials

The following are available online at https://www.mdpi.com/article/10.3390/bios11060192/s1. Figure S1: Cyclic voltammogram of 0.05mM CFN in 0.1M BR buffer solution (pH, 7) on bare GC electrode at scan rates ranging (50–1000) mV/s. Figure S2. Relationship between peak current intensity of 0.05mM CFN and square root of scan rates ranging from 50–1000mV/s. Figure S3. Current response related to number of scan cycles used during electro polymerization of CFN-Py on GC electrode. Figure S4. Comparison of concentration values 25, 40, 55, 70, and 85 µM CFN obtained in the experimental set with GC/MS and DPV on CFN-MIP sensor (n = 3 for each concentration). Table S1. Intra-day and inter-day precision for seven concentrations of CFN using DPV measurements at CFN-MIP sensor. Table S2. Recovery experiments for various concentrations of CFN on CFN-MIP electrode using DPV measurements.
