# Supplementary material for: Computational Design of a Molecularly Imprinted Polymer for the Biomonitoring of the Organophosphorous Metabolite Chlorferron
Source: Biosensors (Basel). 2021 Jun 10;11(6):192. doi: 10.3390/bios11060192 (PMC8227226; doi:10.3390/bios11060192)
Supplement: Supplementary file 1 [file biosensors-11-00192-s001.zip › biosensors-1187019-supplementary.pdf]

biosensors MDPI

Article

Computational Design of a Molecularly Imprinted Polymer for the Biomonitoring of the Organophosphorous Metabolite Chlorferron

Bakhtiyar Qader [1,2], Issam Hussain [3], Mark Baron [2], Rebeca Jiménez-Pérez [2,4], Guzmán Gil-Ramírez [2] and Jose Gonzalez-Rodriguez [2,*]

[1] Sulaimani Medicolegal Institute, Qanat Street, Sulaimani, Sulaymaniyah, 46001, Kurdistan Regional Government, Iraq; bakhtyar88@gmail.com
[2] Joseph Banks Laboratories, School of Chemistry, University of Lincoln, Lincoln LN6 7DL, UK; mbaron@lincoln.ac.uk (M.B.); Rebeca.Jimenez@uclm.es (R.J.-P.); GGilramirez@lincoln.ac.uk (G.G.-R.)
[3] School of Life Sciences, University of Lincoln, Brayford Pool, Lincoln LN6 7TS, UK; ihussain@lincoln.ac.uk
[4] Department of Physical Chemistry, Higher Technical School of Industrial Engineering, University of Castilla-La Mancha, Campus Universitario s/n, 02071 Albacete, Spain
* Correspondence: jgonzalezrodriguez@lincoln.ac.uk

Supplementary Materials:

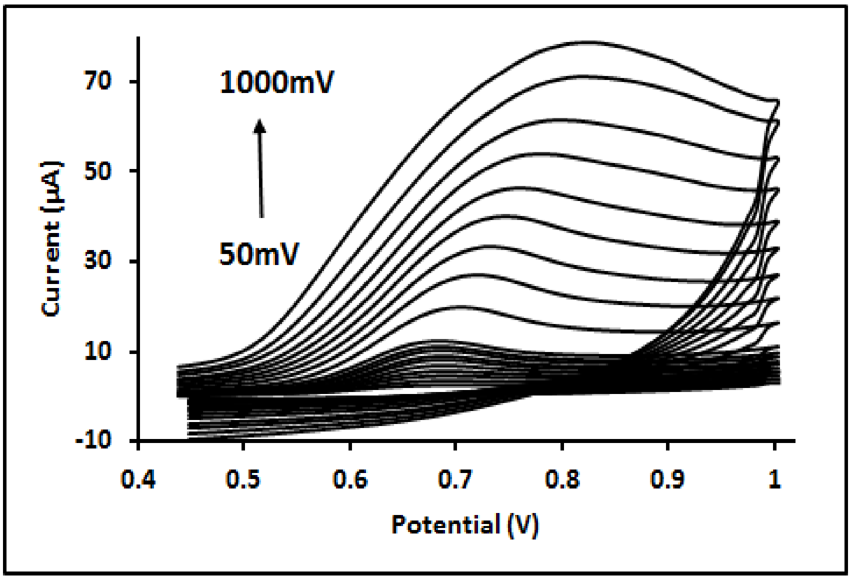

Figure S1. Cyclic voltammogram of 0.05mM CFN in 0.1M BR buffer solution (pH, 7) on bare GC electrode at scan rates ranging (50–1000) mV/s.

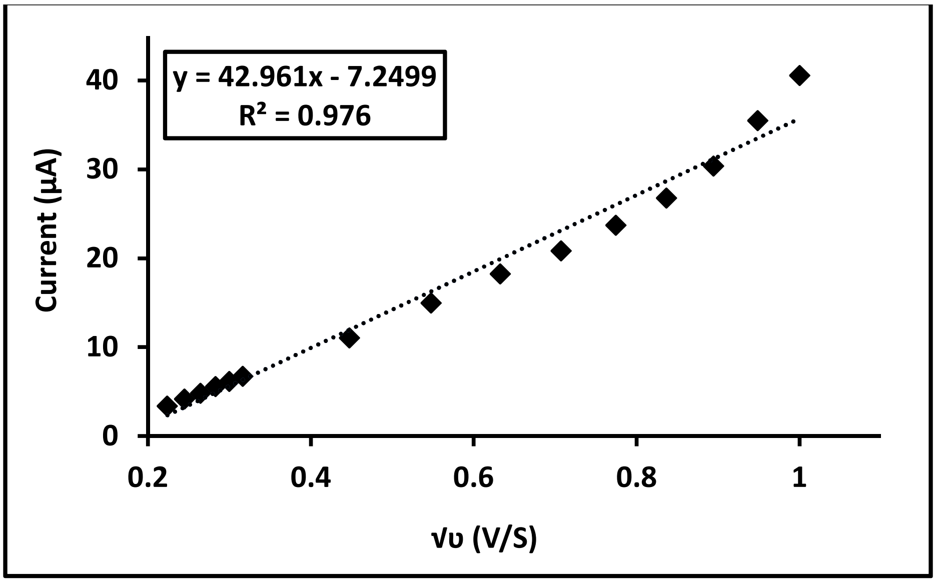

Figure S2. Relationship between peak current intensity of 0.05mM CFN and square root of scan rates ranging from 50–1000 mV/s.

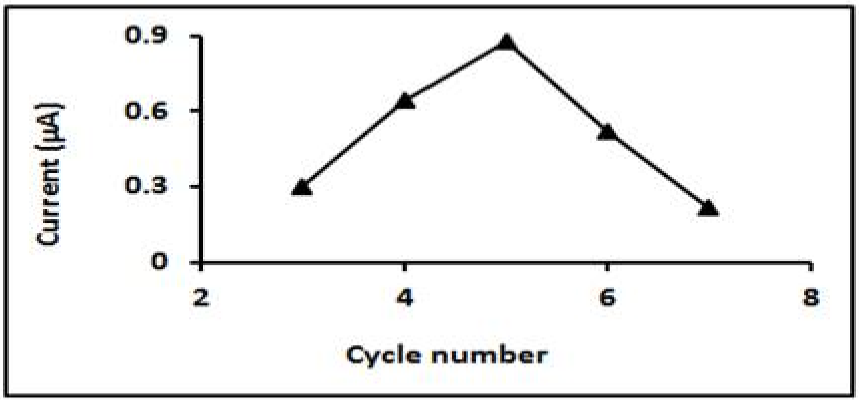

Figure S3. Current response related to number of scan cycles used during electro polymerization of CFN-Py on GC electrode.

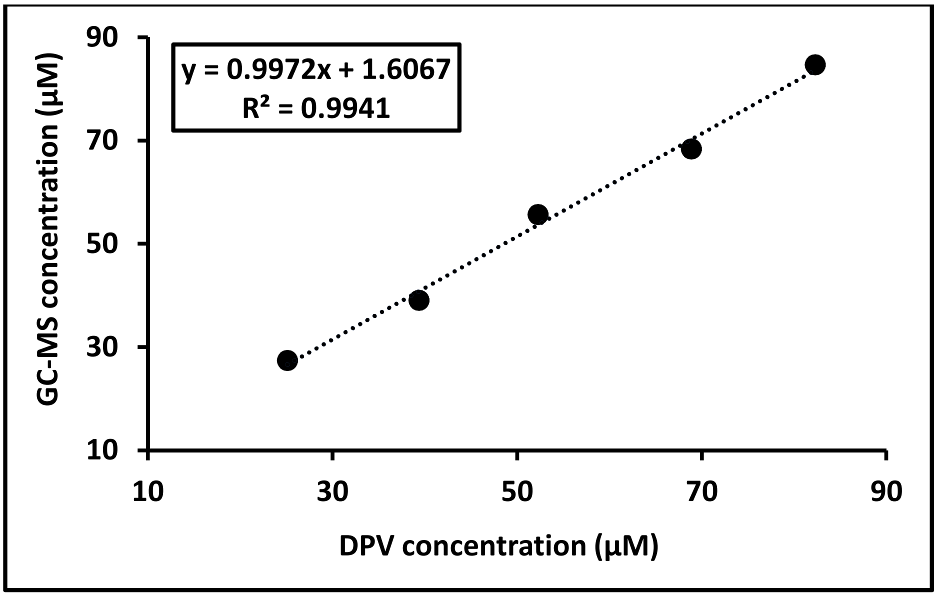

Figure S4. Comparison of concentration values 25, 40, 55, 70, and 85 µM CFN obtained in the experimental set with GC/MS and DPV on CFN-MIP sensor (n = 3 for each concentration).

The samples have been analysed by the two methods. If both methods are equivalent the slope is 1 and the intercept is 0. i.e. y=x. This would indicate both methods produce identical concentration values for the same sample.

Table S1. Intra-day and inter-day precision for seven concentrations of CFN using DPV measurements at CFN-MIP sensor.

Concentration (µM)	Intra-Day Precision		Inter-Day Precision	
	Mean ±SD (µM)	RSD (%)	Mean ±SD (µM)	RDS (%)
2	3.19±0.73	8.75%	2.98±0.73	13.1%
10	13.1±1.6	5.36%	11.6±0.58	10.3%
25	25.1±0.48	4.5%	21.1±0.38	8.17%
40	36.9±0.75	3.04%	36.5±2.9	3.58%
55	48.3±0.35	3.19%	49.8±0.54	4.88%
70	66.1± 0.47	3.13%	68.4±0.62	4.03%
85	91.2± 0.48	2.35%	87.7±0.64	3.22%

Table S2. Recovery experiments for various concentrations of CFN on CFN-MIP electrode using DPV measurements.

Concentration (µM)	Recovered Concentration (N=3)		
	Mean ±SD (µM)	RSD (%)	Recovered Percentage (%)
2	2.27±0.17	11.15%	80.61%
10	10.2±1.4	13.7%	102.02%
25	25.1±1.46	5.83%	100.42%
40	39.35±2.35	5.96%	98.37%
55	52.25±4.65	8.57%	98.64%
70	68.86±6.03	8..75%	98.38%
85	82.3±4.96	6.02%	96.82%
